# The Construction and Analysis of Infiltrating Immune Cell and ceRNA Networks in Diabetic Foot Ulcer

**DOI:** 10.3389/fendo.2022.836152

**Published:** 2022-07-14

**Authors:** Lin Zeng, Pengxiang Zhang, Zebin Fang, Deliang Liu, Huilin Li, Xin Qu, Shufang Chu, Hengxia Zhao, Xuemei Liu, Maosheng Lee

**Affiliations:** ^1^ Fourth Clinical Medical College of Guangzhou University of Chinese Medicine, Shenzhen, China; ^2^ Department of Endocrinology, Shenzhen Traditional Chinese Medicine Hospital, Shenzhen, China

**Keywords:** diabetic foot ulcer, competing endogenous RNA network, infiltrating immune cell, JunB, GATA3, hsa-circ-0049271, hsa-circ-0074559

## Abstract

**Background:**

Diabetic foot ulcer (DFU) is a severe complication characterized by low-grade infectious inflammation and probably associated with specific competitive endogenous RNAs (ceRNAs) and infiltrating immune cells. Nonetheless, no reliable biomarkers are used for detecting infectious inflammation in DFU. Therefore, it is essential to explore potential biomarkers for the accurate diagnosis and treatment of DFU.

**Methods:**

The gene expression profile was retrieved from Gene Expression Omnibus (GEO) database and divided into two groups, namely, standard samples and DFU samples. To establish the ceRNA networks, Gene Ontology (GO) and Kyoto Encyclopedia of Genes and Genomes (KEGG) pathway enrichment analyses were utilized to analyze differential expression genes (DEGs). The cell type identification was achieved by estimating relative subsets of RNA transcripts (CIBERSORT) algorithm to screen-specific immune-infiltrating cells associated with DFU.

**Results:**

A ceRNA network was constructed with 20 differential expression circRNA (DEcircRNAs), 11 differential expression microRNAs (DEmiRNAs), and 9 differential expression mRNAs (DEmRNAs). Functional enrichment analysis demonstrated that DFU was mainly enriched in vascular endothelial growth factor (VEGF) and T-cell receptor signaling. In addition, CIBERSORT estimation indicated that CD8^+^ T cells and Monocytes were significantly related to the expression of IL-6, a DFU-specific infectious inflammation factor.

**Conclusion:**

This study identified that some significant ceRNAs (JUNB, GATA3, hsa-circ-0049271 and hsa-circ-0074559) and infiltrating immune cells (CD8^+^ T cells and monocytes) might be related to DFU infectious inflammation.

## Introduction

It is estimated that, every 20 s, a case of diabetic amputation has been witnessed (1). Although the annual mortality rate of diabetic foot ulcers (DFUs) is 11% (2), several studies have demonstrated that DFU treatment accounts for about one-third of the total cost of diabetes (3). Thus, DFU is not only one of the leading causes of disability and death in diabetic patients but also a significant public health problem, placing a heavy burden on the social and economic development. Furthermore, despite the progress in biological and engineering technologies, functional recovery remains poor due to the limited understanding of DFU pathogenesis. Therefore, exploring the molecular mechanisms of DFU is crucial for developing a therapeutic approach.

Circular RNAs (circRNAs) have a closed ring-like structure that resists the function of RNA extrinsic enzymes (4). Through research and development, circRNAs have gradually become star molecules in non-coding RNA in recent years. Rich in microRNA (miRNA) binding sites, circRNAs entirely relieve the inhibition of miRNA on targeted genes acting as miRNA sponges at the transcription level (5). Furthermore, evidence has demonstrated that circRNAs functioning as competitive endogenous RNA (ceRNAs) are significantly associated with the onset and development of DFU. Wang et al. found that hsa_circ_0084443 is upregulated in DF U and modulates keratinocyte migration and proliferation (6). Liao et al. constructed the ceRNA network of DFU consisting of 8 circRNAs, 11 miRNAs, and 91 mRNAs (7). In addition, immune cell infiltration plays critical role in occurrence and development of DFU. Sawaya et al. found FOMI1 and STAT3, the transcription factors which promote survival of immune cells were inhibited in DFU, which eventually impaired human diabetic wound healing (8). However, few studies focus on the regulatory mechanism of ceRNA and the infiltrating immune cells in DFU.

This study aimed to set up a ceRNA network based on DFU-related ceRNAs to investigate the effect of circRNAs on pathogenesis and treatment of DFU. At first, the sequencing data of mRNA, miRNA, and circRNA from Gene Expression Omnibus (GEO) database were extracted to retrieve differentially expressed mRNAs (DEmRNAs), differentially expressed miRNAs (DEmiRNAs), and differentially expressed circRNAs (DEcircRNAs). Then, a circRNAs–miRNAs–mRNAs network was constructed to explore the roles of circRNAs in DFU. Finally, the immune cell ratios associated with DFU of RNA transcripts (CIBERSORT) algorithm were calculated to elevate the prognosis of DFU depending on the immune cells and ceRNAs types.

## Materials and Methods

### Microarray Data Archives

The microarray data were retrieved from the GEO database. The circRNA, miRNA, and mRNA expression profiles were obtained from GSE114248 (GPL21825 074301 Arraystar Human CircRNA microarray V2, five DFU and five non-DFU samples) (6), GSE84971 (GPL17537 nCounter Human miRNA Expression Assay, V2, three DFU and three non-DFU samples) and GSE68185 (four DFU and four non-DFU samples) (9), GSE80178 (GPL16686 [HuGene-2_0-st] Affymetrix Human Gene 2.0 ST Array [transcript (gene) version], six DFU and three non-DFU tissues) (10), and GSE143735 (11), respectively. The species were *Homo sapiens*.

### Differential Expression Analysis of circRNAs, miRNAs, and mRNAs

The analysis of DEcircRNAs in GSE114248, DEmiRNAs in GSE68185 and GSE84971, and DEmRNAs in GSE80178 and GSE143735 was screened using the limma package in R (12). P-value < 0.05 and |log_2_(fold change, FC)| >1.0 were set as the thresholds of differential genes.

### GO and KEGG Pathway Enrichment Analysis

Gene Ontology (GO) analysis is extensively incorporated to identify the characteristic gene attributes, gene products, and sequences, including biological processes (BP), cell components (CC), and molecular functions (MF) (13). Kyoto Encyclopedia of Genes and Genomes (KEGG) enrichment analysis provides a comprehensive biointerpretation of genomic sequences and information on protein interaction networks (14). This study completed and visualized the GO terms and KEGG pathway enrichment analysis of DEGs using the clusterProfiler V3.14.0 in the R software (15).

### GSEA and GSVA

Gene set enrichment analysis (GSEA) identified genes with statistically significant differences between standard and DFU samples (16). Through referring to gene sets “c5.go.v7.4.entrez.gmt” and “c2.cp.kegg.v7.4.entrez.gmt”, GSE80187 and GSE143735 were enriched by using the R package.

Gene set variation analysis (GSVA) estimates the unsupervised variation of pathway activity population (17). For example, based on gene set “c2.cp.kegg.v7.4.entrez.gmt”, GSVA screened significantly different pathways between standard and DFU samples with P-value < 5% and a false discovery rate < 25%.

### Construction of PPI Network

The protein-protein interaction (PPI) network of the DEmRNAs was developed with the STRING database (http://string-db.org/) (18) on the basis of starBase (19), miRwalk (20), and CirnBank (21) databases. Based on TRRUST (22) and CTD (23) databases, a transcription factors-DEmRNAs-drugs network was constructed. It was then visualized by Cytoscape 3.7.1 software (24).

### Immune Infiltration Analysis

CIBERSORT is a deconvolution algorithm validated on gene expression profiles provided by RNA-sequencing (25). CIBERSORT calculates a P-value for the deconvolution of each sample, which increases confidence in the results. To analyze immune cell proportion in DFU samples, CIBERSORT was employed to evaluate the abundance of 22 human hematopoietic cell phenotypes in GSE80187 and GSE143735. The Wilcoxon test was taken to calculate the difference, and a P-value of less than 0.05 indicated a statistically significant difference.

## Results

### Differentially Expressed DEGs, DEMs, and DECs

**Figure 1:**
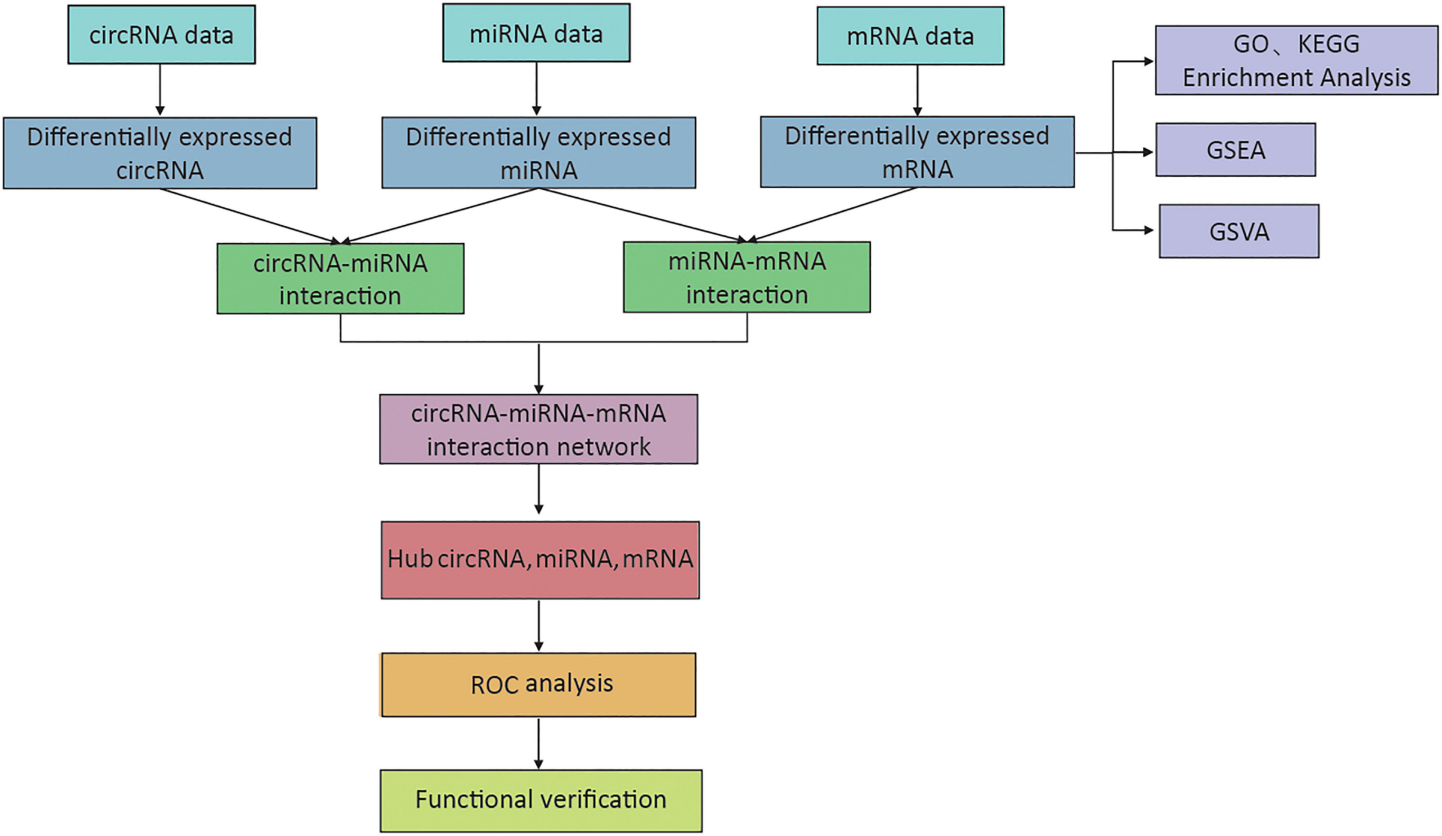
The flowchart of the whole research process.

The expression of circRNA, miRNA, and mRNA in DFU samples was determined by microarray analysis (**Supplementary Table 1**). The methods and results of our study are shown in **Figure 1**.

**Figure 2:**
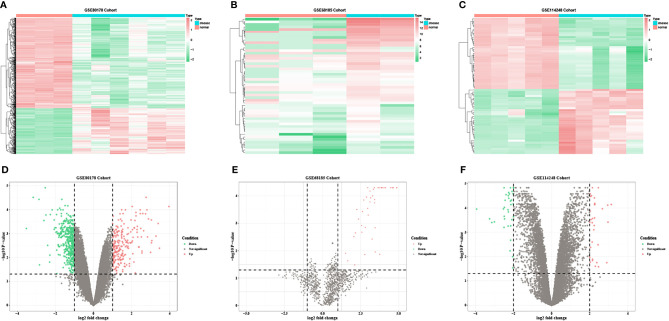
The heatmap and volcano plots of DEmRNAs in GSE80178, DEmiRNAs in GSE68185, and DEcircRNAs in GSE114248. **(A)** Heatmap of DEmRNAs in GSE80178. **(B)** Heatmap of DEmiRNAs in GSE68185. **(C)** Heatmap of DEcircRNAs in GSE114248. **(D)** Volcano plot of DEmRNAs in GSE80178. **(E)** Volcano plot of DEmiRNAs in GSE68185. **(F)** Volcano plot of DEcircRNAs in GSE114248. Data points in red and green represent upregulated and downregulated, respectively.

**Figure 3:**
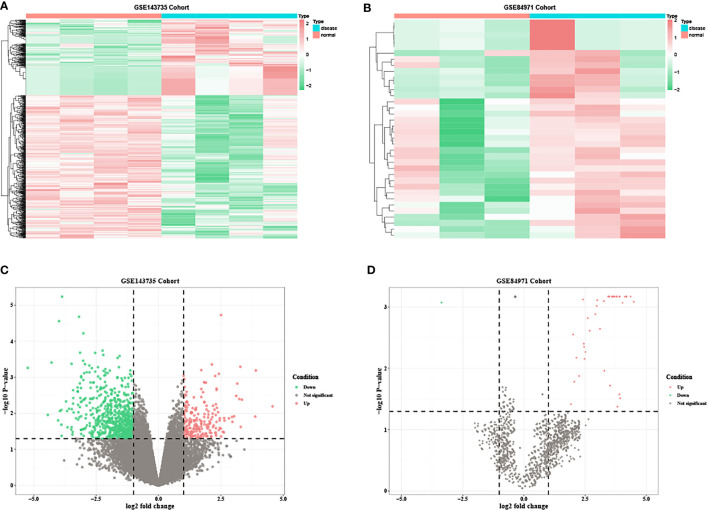
The heatmap and volcano plots of DEmRNAs in GSE143735 and DEmiRNAs in GSE84971. **(A)** Heatmap of DEmRNAs in GSE143735. **(B)** Heatmap of DEmiRNAs in GSE84971. **(C)** Volcano plot of DEmRNAs in GSE143735. **(D)** Volcano plot of DEmiRNAs in GSE84971.

Based on the predetermined threshold (|log_2_FC| > 1.0 and adjusted P < 0.05), 718 DEmRNAs were identified (241 upregulated and 477 downregulated) in GSE80187, 1,070 DEmRNAs (370 upregulated and 700 downregulated) in GSE143735, 52 DEmiRNAs (52 upregulated) in GSE68185, 37 DEmiRNAs (36 upregulated and one downregulated) in GSE84971, and 67 DEcircRNAs (32 upregulated and 35 downregulated) in GSE114248. Subsequently, the heatmap and volcano plot of DEmRNAs in GSE80187, DEmiRNAs in GSE68185, and DEcircRNAs in GSE114248 are shown in **Figure 2**. Meanwhile, DEmRNAs in GSE143735 and DEmiRNAs in GSE84971 are depicted in **Figure 3**.

### GO and KEGG Functional Enrichment Analysis

**Figure 4:**
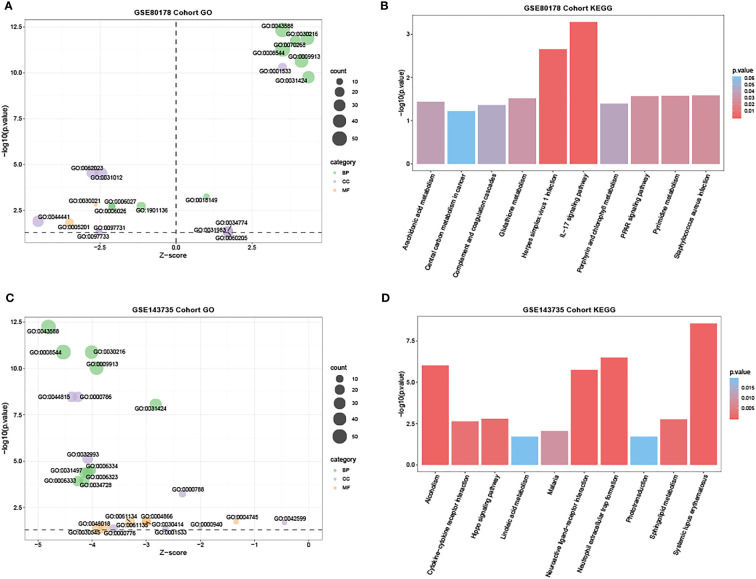
GO and KEGG functional enrichment analysis in GSE80178 and GSE143735. **(A)** The bubble chart of the GO terms, including BP, MF, and CC, was significantly enriched in GSE80178. Green bubbles represent BP; purple bubbles represent MF; orange bubbles represent CC. **(B)** Histogram of the top 10 KEGG pathway enrichment in GSE80178. **(C)** Bubble chart of the GO terms, including BP, MF, and CC, was significantly enriched in GSE143735. The green bubble represents BP; the purple bubble represents MF; the orange bubble represents CC. **(D)** Histogram of the top 10 KEGG pathway enrichment in GSE143735.

Based on the DAVID database, GO enrichment analysis revealed the top 21 enriched GO terms of the DEmRNAs in GSE80178 (**Supplementary Table 2**). In BP, DEmRNAs were significantly enriched in skin development, keratinocyte differentiation, cornification, epidermis development, and epidermal cell differentiation. MF also possessed some related enriched terms such as extracellular matrix structural constituent and conferring compression resistance. In CC, DEmRNAs were mainly enriched in the cornified envelope and extracellular matrix (**Figure 4A**). KEGG pathway analysis indicated that DEmRNAs were primarily enriched in the interleukin-17 (IL-17) signaling pathway, herpes simplex virus-1 infection, *Staphylococcus aureus* infection, peroxisome proliferator-activated receptor (PPAR) signaling pathway, and arachidonic acid metabolism (**Figure 4B** and **Supplementary Table 3**).

Meanwhile, in GSE143735, GO analysis enrichment revealed the top 25 enriched GO terms of the DEmRNAs (**Supplementary Table 4**). In BP, DEmRNAs were significantly enriched in skin and epidermis development and keratinocyte and epidermal cell differentiation. Specific enriched terms like activities of endopeptidase inhibitor, retinol dehydrogenase, endopeptidase regulator, and peptidase inhibitor were related to MF (**Figure 4C**). In CC, DEmRNAs were primarily enriched in DNA packaging, protein-DNA complexes, and nucleosome. KEGG pathway analysis revealed that DEmRNAs were mainly enriched in neutrophil extracellular trap formation, neuroactive ligand-receptor and cytokine-cytokine receptor interactions, and hippo signaling pathway (**Figure 4D** and **Supplementary Table 5**).

### GSEA and GSVA Analysis

**Figure 5:**
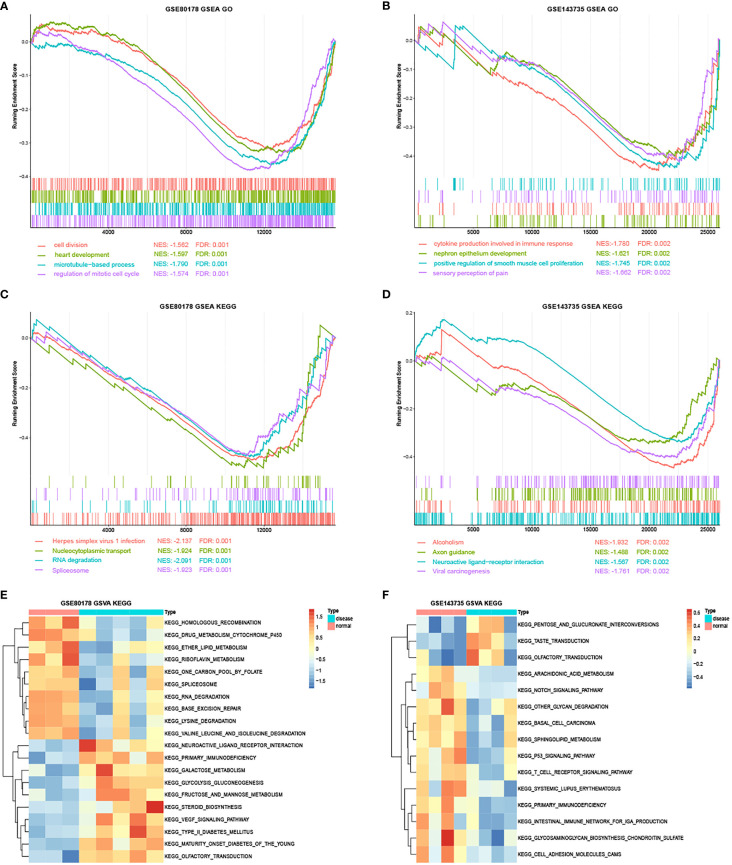
The GSEA and GSVA analysis in GSE80178 and GSE143735. **(A)** Top four GO annotations with a P-value in GSE80178 through GSEA analysis. **(B)** Top four GO annotations with a P-value in GSE143735 through GSEA analysis. **(C)** Top four KEGG pathway with a P-value in GSE80178 through GSEA analysis. **(D)** Top four KEGG pathway with a P-value in GSE143735 through GSEA analysis. **(E)** Top 20 KEGG pathway with a P-value in GSE80178 through GSVA analysis. **(F)** Top 20 KEGG pathway with a P-value in GSE143735 through GSVA analysis.

GSEA depicted that the most enriched gene sets in GSE80187 were positively correlated with BP, such as cell division, regulation of mitotic cell cycle and phase transition, and signaling pathways in herpes simplex virus-1 infection, nucleocytoplasmic transport, and RNA degradation (**Figures 5A, C** and **Supplementary Table 6**). Moreover, enriched gene sets were positively correlated with BP in GSE143735, such as cytokine production in immune response, positive regulation of smooth muscle cell proliferation, and signaling pathways in neuroactive ligand-receptor interaction, axon guidance, and viral carcinogenesis (**Figures 5B, D** and **Supplementary Table 7**).

GSVA indicated that signaling pathways, such as RNA degradation, vascular endothelial growth factor (VEGF), homologous recombination, glycosaminoglycan biosynthesizes chondroitin sulfate, and T-cell receptors were significantly more enriched in DFU samples than standard samples (**Figures 5E, F** and **Supplementary Tables 8, 9**).

### Construction of the circRNAs-miRNA-mRNA Network

**Figure 6:**
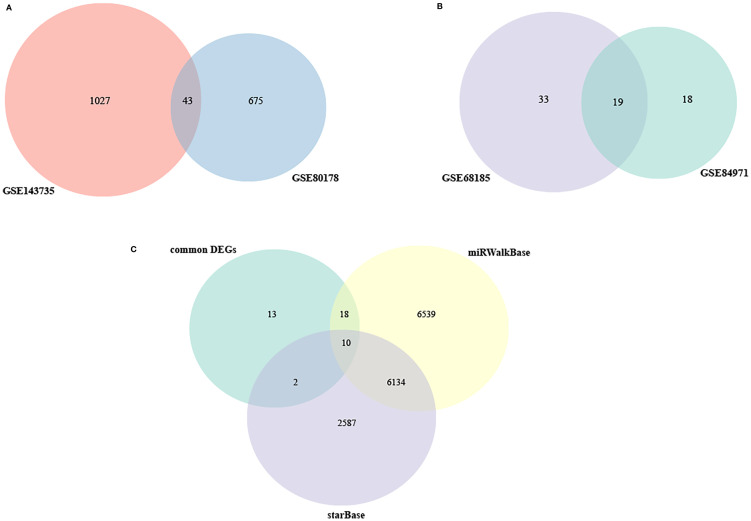
The Venn diagram of DEGs and DEMs. **(A)** Venn diagram of DEGs in GSE80178 and GSE143735. **(B)** Venn diagram of DEMs in GSE68185 and GSE84971. **(C)** Venn diagram of DEMs in GSE80178, GSE143735, and GSE84971.

**Figure 7:**
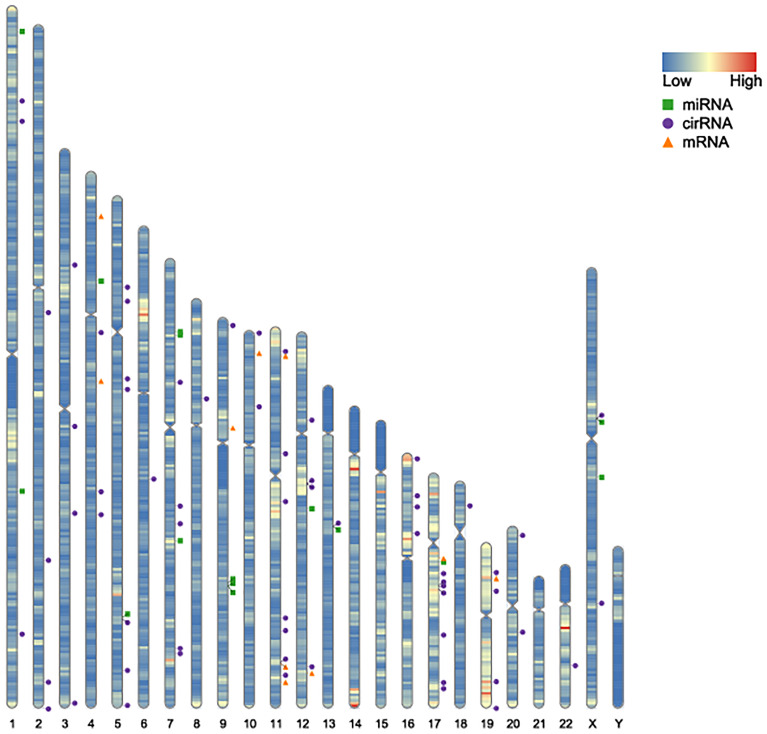
Location on the chromosomes of DEGs, DEMs, and DECs. The orange triangle represents the chromosomal location of DEGs in GSE80178 and GSE143735. The green block represents the chromosomal location of DEMs in GSE68185 and GSE84971. The blue spot represents the chromosomal location of DECs in GSE114248.

A total of 43 common DEGs from GSE80187 and GSE143735 (**Figure 6A**) and 19 common DEMs from GSE68185 and GSE84971 were screened (**Figure 6B**). First, based on the miRWalk and starBase databases, 10 overlapping DEGs were screened, including EREG, JUNB, OASL, FGFBP1, BLMH, USP2, CDON, AMPD3, CNTNAP3, and GATA3 (**Figure 6C**). Then, the ideogram function was used to draw the location of 10 overlapping DEGs, 19 overlapping DEMs, and 67 overlapping DECs on the chromosomes (**Figure 7**) (26).

**Figure 8:**
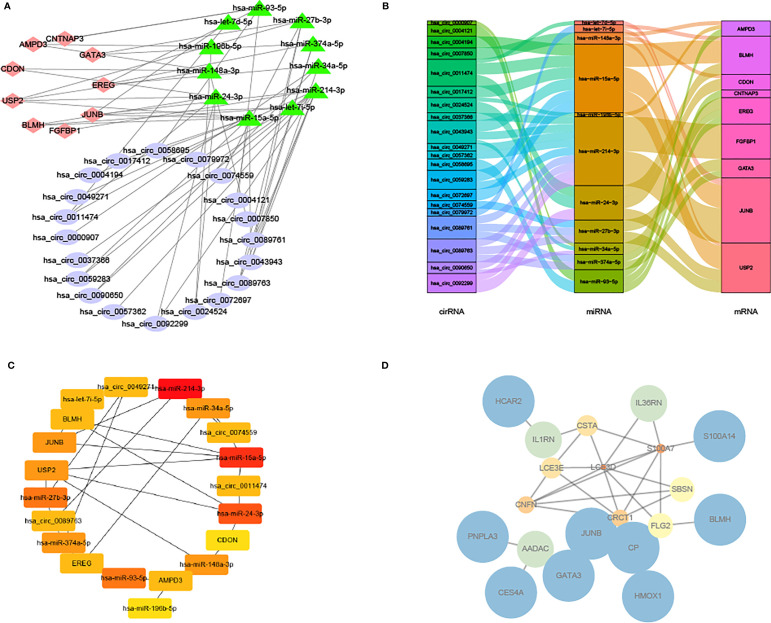
Network and PPI network of circRNA-miRNA-mRNA. **(A)** Network of circRNA-miRNA-mRNA. **(B)** Sankey diagram of the relationship between circRNA-miRNA-mRNA. **(C)** Network of significant circRNA-miRNA-mRNA. **(D)** PPI network of DEGs in GSE80178 and GSE143735.

Next, the circRNA-miRNA-mRNA network (**Figure 8A**) was constructed according to the Sankey diagram (**Figure 8B**). It was found that circRNA mainly corresponded to hsa-miR-24–3p and hsa-miR-214–3p, and miRNA was specifical compared to JUNB and BLMH. Then, a significant circRNA-miRNA-mRNA network was constructed (**Figure 8C**); meanwhile, the PPI network of DEGs was visualized in GSE80178 and GSE143735 and screened by the STRING database (**Figure 8D**).

### ROC Analysis

**Figure 9:**
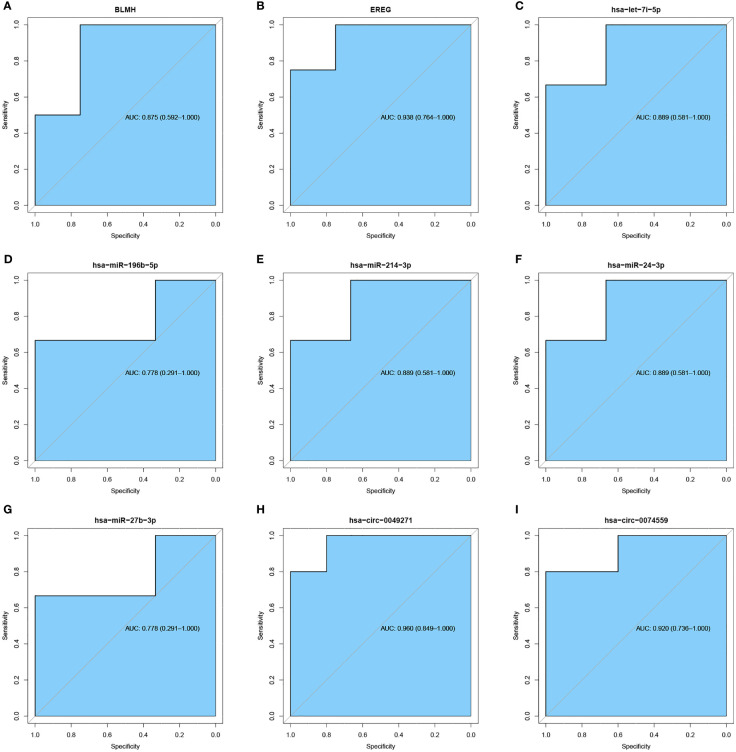
ROC curve of significant mRNA, miRNA, and circRNA. **(A, B)** ROC curve of significant mRNA. **(C–G)** ROC curve of significant miRNA. **(H, I)** ROC curve of significant circRNA. The blue areas represent AUC.

ROC analysis evaluated the accuracy of crucial molecules in distinguishing whether a sample is DFU or regular by calculating AUC. The diagnostic criteria of DFU are referenced in the previous study (27). It was observed that circRNA, like hsa-circ-0049271 and hsa-circ-0074559; miRNAs, such as hsa-let-7i-5p, hsa-miR-24–3p, and hsa-miR-214–3p; and genes, including BLMH and EREG, had the classification predicting values (**Figures 9A–I**).

### Immune Infiltration Analysis

**Figure 10:**
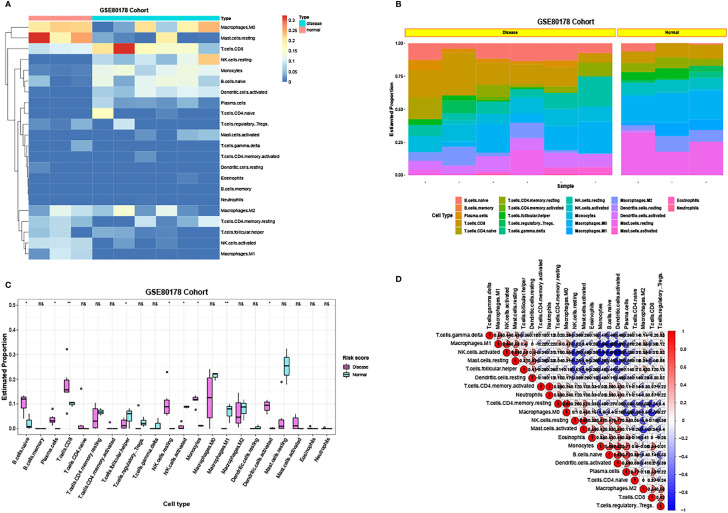
The ratio of immune cells between standard samples and DFU samples. **(A)** Heatmap of the ratio of 22 immune cells between standard and DFU samples in GSE80178. **(B)** Histogram of the ratio of 22 immune cells between standard and DFU samples in GSE80178. **(C)** Box diagram of the ratio of 22 immune cells between standard and DFU samples in GSE80178. **(D)** Correlation of 22 immune cells in GSE80178.

Using CIBERSORT, the ratio of 22 immune cells between standard and DFU samples was estimated (**Figure 10A**). In GSE80178, the proportions of immune cells are individual and have group difference; and the balance of T cells was higher in DFU samples, whereas macrophages were higher in standard samples (**Figure 10B**).

Then, the proportion of these immune cells between standard and DFU samples was compared. The abundance of B.cells.naïve, Plasma.cells, T.cells.CD8, Macrophages.M1, T.cells.follicular.helper, NK.cells.resting, NK.cells.activated, Dendritic.cells.activated, and monocytes showed a significant statistical difference (**Figure 10C**). Next, the correlation of the 22 immune cells was visualized (**Figure 10D**).

**Figure 11:**
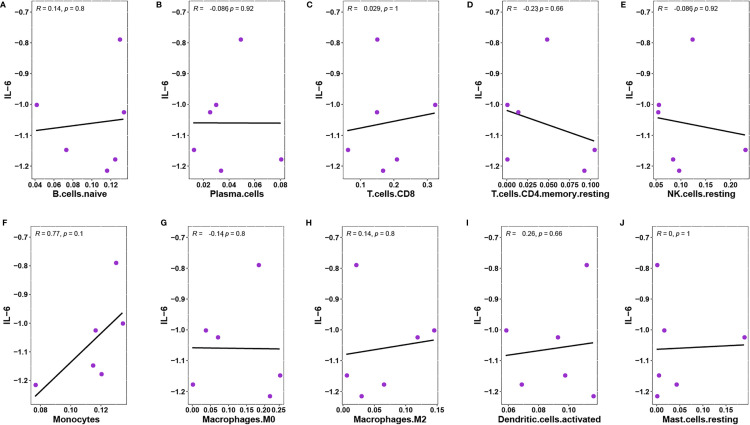
**(A)** Pearson’s correlation between IL-6 and B.cells.naive. **(B)** Pearson’s correlation between IL-6 and Plasma.cells. **(C)** Pearson’s correlation between IL-6 and T.cells.CD8. **(D)** Pearson’s correlation between IL-6 and T.cells.CD4.memory.resting. **(E)** Pearson’s correlation between IL-6 and NK.cells.resting. **(F)**Pearson’s correlation between IL-6 and Monocytes. **(G)** Pearson’s correlation between IL-6 and Macrophages.M0. **(H)** Pearson’s correlation between IL-6 and Macrophages.M2. **(I)** Pearson’s correlation between IL-6 and Dendritic.cells.activated. **(J)** Pearson’s correlation between IL-6 and Mast.cells.resting.

Obvious studies have demonstrated that IL-6 seems to be an inflammatory marker in the discrimination of infected DFU (28, 29). Through a Pearson’s analysis, it was observed that the proportion of T.cells.CD8 and monocytes were positively correlated with the expression of IL-6, whereas T.cells.CD4.memory.resting and NK.cells.resting were negatively correlated with IL-6 expression (**Figures 11A–J**).

### Network Analysis of the Intersection of the Target Genes of mRNA and miRNA

**Figure 12:**
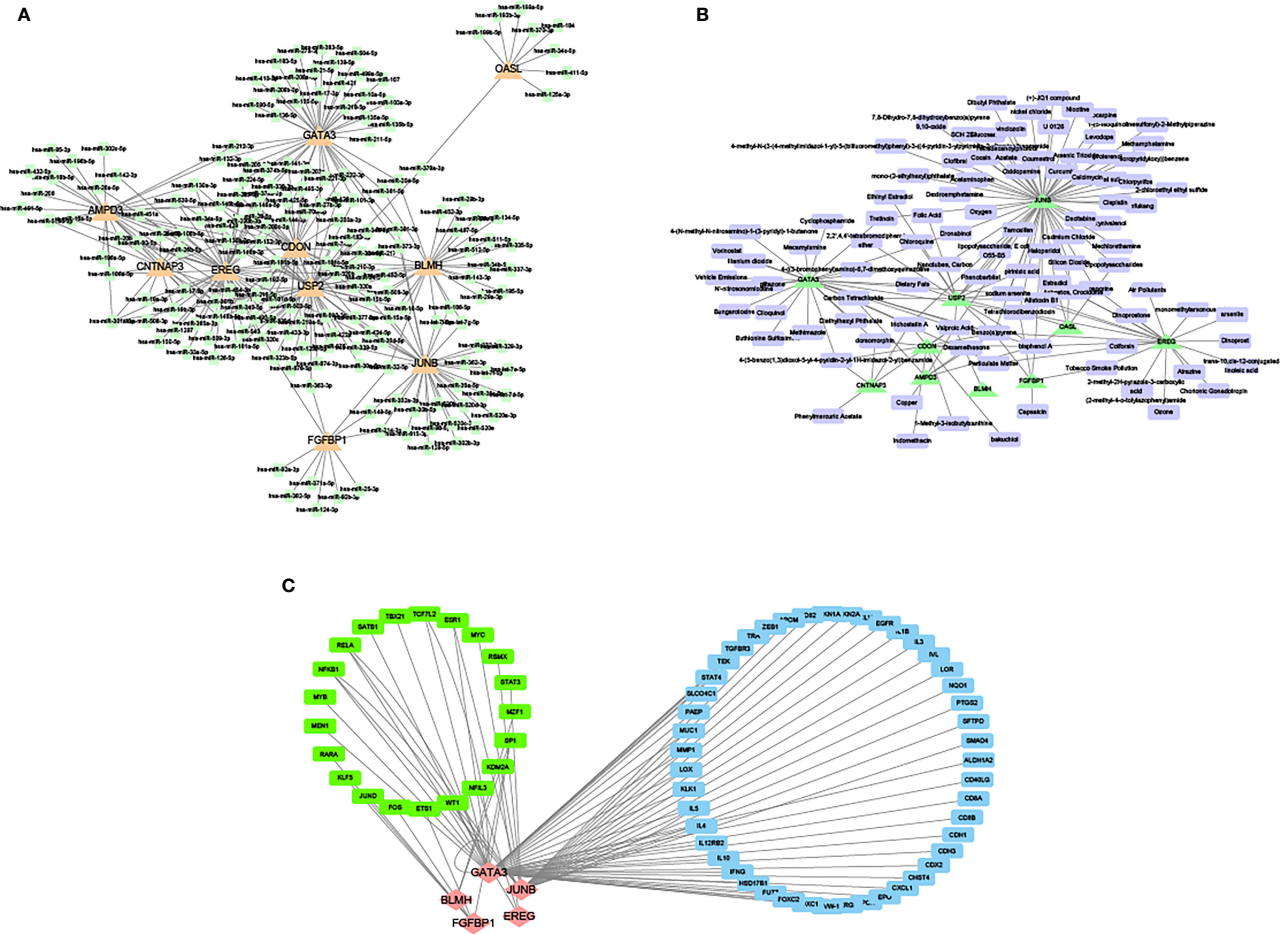
Network of miRNA and mRNA. **(A)** The network between overlapping mRNA and miRNA. Orange triangles represent overlapping mRNA. Green blocks represent miRNA. **(B)** The network between overlapping and targeted drugs. Green triangles represent overlapping mRNA. Purple blocks represent targeted drugs. **(C)** The network between overlapping mRNA and transcription factors. Red rhombuses express mRNA. Green blocks represent transcription factors that regulate mRNA. Blue blocks represent genes that are regulated when mRNA is a transcription factor.

The miRNA corresponding to the common DEGs was predicted and the mRNA-miRNA network based on the starBase databases was constructed (**Figure 12A**). Then, the drugs targeted by overlapping miRNA were retrieved and the gene-drug network based on the Comparative Toxicogenomics Database database was created (**Figure 12B**).

Meanwhile, the mRNA regulated by overlapping mRNA and transcription factors from the TRRUST database was retrieved and the network between mRNA and transcription factors was developed (**Figure 12C**).

It was observed that JUNB and GATA3 were higher in the degree of connectivity in the gene miRNA transcription factor drug network, suggesting that these genes may be important targets for DFU.

## Discussion

Advanced research using next-generation sequencing technology has indicated the role of circRNA-associated ceRNA network in DFU. For example, studies had demonstrated that circRNA cPWWP2A interacted with miR-579 to upregulate the expression of angiopoietin 1, occludin 1, and SIRT1 acting as a ceRNA, consequently promoting retinal vascular dysfunction (30, 31). Liao et al. constructed the ceRNA network of DFU consisting of 8 circRNAs, 11 miRNAs, and 91 mRNAs, and GO analysis showed that hub genes including BCL2, CDND1, and SMAD4 are potential diagnostic biomarkers in DFU (7). At the same time, Qian et al (32). identified 1,192 DEGs in the GSE7014 dataset (900 upregulated and 292 downregulated) and 1,176 DEGs in the GSE29221 dataset (257 upregulated and 919 downregulated). However, it is unclear how the circRNA-associated ceRNA network and infiltrating immune cell are connected to the pathogenesis of DFU. Therefore, the construction and analysis of infiltrating immune cell and ceRNA networks have to be constructed to deepen the understanding of the molecular mechanism of DFU. The study created a ceRNA regulatory network in DFU consisting of 20 DEcircRNAs, 11 DEmiRNAs, and 8 DEmRNAs.

Angiogenesis extends blood vessels through vascular branching. DFU has been reported to be associated with poor angiogenesis in granulation tissues. Therefore, wound tissue angiogenesis could be a viable therapy for DFU. The transcription factors of the activator protein 1 (AP-1) are involved in the cell cycle and cell development, eventually regulating angiogenesis and vascular growth (33). Several members of the AP-1 family and JUNB are associated with embryonic fibroblast cell proliferation (34) and T-cell programming (35). Endothelial cells play a significant role in maintaining the function of blood vessels. JUNB knockdown attenuated the migration instead of the proliferation of HUVEC, primarily indicating the regulated migration of endothelial cells induced by JUNB and the eventual participating in angiogenesis of JUNB (33). The study similarly showed that JUNB might act as the target gene for DFU.

It was found that circRNAs (hsa-circ-0049271 and hsa-circ-0074559) and miRNAs (hsa-let-7i-5p, hsa-miR-24–3p, and hsa-miR-214–3p) were identified as significant predictors to distinguish DFU from standard samples. Functional enrichment analysis demonstrated DFU-related pathways, including VEGF and T-cell receptor signaling. A recent study showed that hsa-circ-0049271 was involved in cell proliferation (36). Guo et al. demonstrated that hsa-miR-24–3p expression is significantly higher in the vitreous of diabetic retinopathy (37), establishing the association of hsa-mir-24–3p with angiogenesis. The VEGF signaling pathway regulated angiogenesis in embryonic skin and blood vessel formation as an upstream JUNB regulator, eventually controlling endothelial cell proliferation, survival, and migration to develop blood vessels (38). CIBERSORT estimation indicated that CD8^+^ T cells and monocytes were related to the expression of IL-6. Therefore, hsa-circ-0049271/hsa-mir-24–3p/JUNB is supposedly the crucial axis for DFU angiogenesis and infectious inflammation.

However, the study had some limitations. First, the data collected from public databases were limited because of a lack of clinical factors, which could factor in potential errors and affect the reliability. Therefore, more experimental evidence included is required to increase the statistical power and authenticate the association between circRNA-miRNA-mRNA network and immune cells in DFU. In addition, the heterogeneity of DFU-related immune microenvironment was not analyzed. Furthermore, the specific mechanism of immune cells was not studied in detail. Therefore, future studies with single-cell sequencing are warranted to achieve more reliable outcomes. Last, the study did not analyze the direct mechanisms among ceRNA, filtrating immune cells, and cellular communication of DFU. Therefore, modern techniques like qRT-PCR, immunofluorescence, immunohistochemistry, and Western blotting are needed to detect the relationship among hsa-circ-0049271, hsa-mir-24–3p, JUNB, CD8^+^ T cells, and monocytes.

In summary, the study aimed to explore the biological functions and pathways involved in the development of DFU. It was identified that two circRNAs, three miRNAs, two hub genes, and nine immune cells are highly associated with the diagnosis and treatment through PPI network and ROC curve analyses. Furthermore, GO and KEGG enrichment analyses demonstrated that the VEGF signaling pathway, smooth muscle cell proliferation, and T-cell receptor signaling pathway could be potential targets for DFU treatment.

## Data Availability Statement

The original contributions presented in the study are included in the article/**Supplementary Material**. Further inquiries can be directed to the corresponding author.

## Author Contributions

LZ and PZ contributed equally to this work. All authors contributed to the article and approved the submitted version.

## Funding

National Natural Science Foundation of China, Grant/Award Numbers: 81704002.

## Conflict of Interest

The authors declare that the research was conducted in the absence of any commercial or financial relationships that could be construed as a potential conflict of interest.

## Publisher’s Note

All claims expressed in this article are solely those of the authors and do not necessarily represent those of their affiliated organizations, or those of the publisher, the editors and the reviewers. Any product that may be evaluated in this article, or claim that may be made by its manufacturer, is not guaranteed or endorsed by the publisher.
