# Supplementary material for: The Construction and Analysis of Infiltrating Immune Cell and ceRNA Networks in Diabetic Foot Ulcer
Source: Front Endocrinol (Lausanne). 2022 Jul 14;13:836152. doi: 10.3389/fendo.2022.836152 (PMC9329527; doi:10.3389/fendo.2022.836152)
Supplement: Supplementary file 1 [file Table_1.docx]

**Supplementary information**

**Table 1 Basic information of the five microarray datasets from GEO**

| Data source | Platform | Author | Year | Region | Sample size | Detected RNA type |
| --- | --- | --- | --- | --- | --- | --- |
| GSE80178 | GPL16686 |  |  |  |  | mRNA |
| GSE143735 | GPL11154 |  |  |  |  | mRNA |
| GSE68185 | GPL17537 |  |  |  |  | miRNA |
| GSE84971 | GPL17537 |  |  |  |  | miRNA |
| GSE114248 | GPL21825 |  |  |  |  | circRNA |

**Table 2. The GO term enrichment of GSE80178**

| **Category** | **ID** | **Description** | **p-value** |
| --- | --- | --- | --- |
| BP | GO:0043588 | skin development | 4.61E-13 |
| BP | GO:0030216 | keratinocyte differentiation | 1.30E-12 |
| BP | GO:0070268 | cornification | 1.88E-12 |
| BP | GO:0008544 | epidermis development | 5.34E-12 |
| BP | GO:0009913 | epidermal cell differentiation | 2.25E-11 |
| BP | GO:0031424 | keratinization | 1.74E-10 |
| BP | GO:0018149 | peptide cross-linking | 0.00054 |
| BP | GO:0006027 | glycosaminoglycan catabolic process | 0.00180 |
| BP | GO:1901136 | carbohydrate derivative catabolic process | 0.00194 |
| BP | GO:0006026 | aminoglycan catabolic process | 0.00200 |
| MF | GO:0030021 | extracellular matrix structural constituent conferring compression resistance | 0.00151 |
| MF | GO:0005201 | extracellular matrix structural constituent | 0.01494 |
| CC | GO:0001533 | cornified envelope | 5.01E-11 |
| CC | GO:0062023 | collagen-containing extracellular matrix | 2.59E-05 |
| CC | GO:0031012 | extracellular matrix | 3.20E-05 |
| CC | GO:0044441 | ciliary part | 0.012149 |
| CC | GO:0034774 | secretory granule lumen | 0.041282 |
| CC | GO:0097733 | photoreceptor cell cilium | 0.048152 |
| CC | GO:0060205 | cytoplasmic vesicle lumen | 0.048152 |
| CC | GO:0031983 | vesicle lumen | 0.048152 |
| CC | GO:0097731 | 9+0 non-motile cilium | 0.048152 |

**Table 3. KEGG enrichment of GSE80178**

| **Category** | **ID** | **Description** | **p-value** |
| --- | --- | --- | --- |
| KEGG_PATHWAY | hsa04657 | IL-17 signaling pathway | 0.00052 |
| KEGG_PATHWAY | hsa05168 | Herpes simplex virus 1 infection | 0.00220 |
| KEGG_PATHWAY | hsa05150 | Staphylococcus aureus infection | 0.02600 |
| KEGG_PATHWAY | hsa00240 | Pyrimidine metabolism | 0.02658 |
| KEGG_PATHWAY | hsa03320 | PPAR signaling pathway | 0.02711 |
| KEGG_PATHWAY | hsa00480 | Glutathione metabolism | 0.03038 |
| KEGG_PATHWAY | hsa00590 | Arachidonic acid metabolism | 0.03670 |
| KEGG_PATHWAY | hsa00860 | Porphyrin and chlorophyll metabolism | 0.04017 |
| KEGG_PATHWAY | hsa04610 | Complement and coagulation cascades | 0.04347 |
| KEGG_PATHWAY | hsa05230 | Central carbon metabolism in cancer | 0.04922 |

**Table 4. The GO term enrichment of GSE143735**

| **Category** | **ID** | **Description** | **p-value** |
| --- | --- | --- | --- |
| BP | GO:0043588 | skin development | 5.94E-13 |
| BP | GO:0008544 | epidermis development | 1.31E-11 |
| BP | GO:0030216 | keratinocyte differentiation | 1.31E-11 |
| BP | GO:0009913 | epidermal cell differentiation | 9.47E-11 |
| BP | GO:0031424 | keratinization | 8.92E-09 |
| BP | GO:0006334 | nucleosome assembly | 3.07E-05 |
| BP | GO:0006323 | DNA packaging | 4.13E-05 |
| BP | GO:0031497 | chromatin assembly | 4.13E-05 |
| BP | GO:0006333 | chromatin assembly or disassembly | 0.000119 |
| BP | GO:0034728 | nucleosome organization | 0.000122 |
| MF | GO:0004866 | endopeptidase inhibitor activity | 0.018287 |
| MF | GO:0004745 | retinol dehydrogenase activity | 0.018287 |
| MF | GO:0061135 | endopeptidase regulator activity | 0.018287 |
| MF | GO:0030414 | peptidase inhibitor activity | 0.018287 |
| MF | GO:0061134 | peptidase regulator activity | 0.018287 |
| MF | GO:0048018 | receptor ligand activity | 0.039002 |
| MF | GO:0030545 | receptor regulator activity | 0.045368 |
| CC | GO:0044815 | DNA packaging complex | 3.42E-09 |
| CC | GO:0000786 | nucleosome | 3.42E-09 |
| CC | GO:0032993 | protein-DNA complex | 7.19E-06 |
| CC | GO:0000788 | nuclear nucleosome | 0.000566 |
| CC | GO:0042599 | lamellar body | 0.019984 |
| CC | GO:0000940 | condensed chromosome outer kinetochore | 0.042954 |
| CC | GO:0000776 | kinetochore | 0.04349 |
| CC | GO:0001533 | cornified envelope | 0.04349 |

**Table 5. KEGG enrichment of GSE143735**

| **Category** | **ID** | **Description** | **p-value** |
| --- | --- | --- | --- |
| KEGG_PATHWAY | hsa05322 | Systemic lupus erythematosus | 2.74E-09 |
| KEGG_PATHWAY | hsa04613 | Neutrophil extracellular trap formation | 3.16E-07 |
| KEGG_PATHWAY | hsa05034 | Alcoholism | 9.44E-07 |
| KEGG_PATHWAY | hsa04080 | Neuroactive ligand-receptor interaction | 1.81E-06 |
| KEGG_PATHWAY | hsa04390 | Hippo signaling pathway | 0.001611 |
| KEGG_PATHWAY | hsa00600 | Sphingolipid metabolism | 0.001762 |
| KEGG_PATHWAY | hsa04060 | Cytokine-cytokine receptor interaction | 0.002304 |
| KEGG_PATHWAY | hsa05144 | Malaria | 0.00889 |
| KEGG_PATHWAY | hsa00591 | Linoleic acid metabolism | 0.019556 |
| KEGG_PATHWAY | hsa04744 | Phototransduction | 0.019556 |

**Table 6. GSEA analysis of GSE80178**

| **Category** | **ID** | **Description** | **NES** | **FDR** |
| --- | --- | --- | --- | --- |
| BP | GO:0007017 | microtubule-based process | -1.7902 | 0.001159 |
| BP | GO:0051301 | cell division | -1.56206 | 0.001164 |
| BP | GO:0007346 | regulation of mitotic cell cycle | -1.5735 | 0.001166 |
| BP | GO:0007507 | heart development | -1.59657 | 0.00117 |
| BP | GO:0044770 | cell cycle phase transition | -1.51444 | 0.0012 |
| BP | GO:0120031 | plasma membrane-bounded cell projection assembly | -2.07857 | 0.001211 |
| BP | GO:0030031 | cell projection assembly | -2.06443 | 0.001212 |
| BP | GO:0016569 | covalent chromatin modification | -1.87282 | 0.001224 |
| BP | GO:0006281 | DNA repair | -2.03994 | 0.001225 |
| KEGG_PATHWAY | hsa05168 | Herpes simplex virus 1 infection | -2.13714 | 0.001253 |
| KEGG_PATHWAY | hsa03040 | Spliceosome | -1.92295 | 0.001383 |
| KEGG_PATHWAY | hsa03013 | Nucleocytoplasmic transport | -1.92425 | 0.001391 |
| KEGG_PATHWAY | hsa03018 | RNA degradation | -2.09091 | 0.00155 |
| KEGG_PATHWAY | hsa04950 | Maturity onset diabetes of the young | 2.044191 | 0.002304 |
| KEGG_PATHWAY | hsa04370 | VEGF signaling pathway | 1.919945 | 0.002494 |
| KEGG_PATHWAY | hsa04625 | C-type lectin receptor signaling pathway | 1.932957 | 0.002747 |
| KEGG_PATHWAY | hsa04380 | Osteoclast differentiation | 1.863918 | 0.003077 |
| KEGG_PATHWAY | hsa04932 | Non-alcoholic fatty liver disease | 2.033251 | 0.003135 |
| KEGG_PATHWAY | hsa04657 | IL-17 signaling pathway | 2.661545 | 0.003205 |

**Table 7. GSEA analysis of GSE143735**

| **Category** | **ID** | **Description** | **NES** | **FDR** |
| --- | --- | --- | --- | --- |
| BP | GO:0072009 | nephron epithelium development | -1.62124 | 0.001873 |
| BP | GO:0002367 | cytokine production involved in immune response | -1.78061 | 0.001876 |
| BP | GO:0019233 | sensory perception of pain | -1.66159 | 0.001876 |
| BP | GO:0048661 | positive regulation of smooth muscle cell proliferation | -1.74516 | 0.001876 |
| BP | GO:0048675 | axon extension | -1.71521 | 0.001876 |
| BP | GO:0007093 | mitotic cell cycle checkpoint | -1.96782 | 0.00188 |
| BP | GO:0014074 | response to purine-containing compound | -1.72547 | 0.00188 |
| BP | GO:0071772 | response to BMP | -1.68986 | 0.00188 |
| BP | GO:0071773 | cellular response to BMP stimulus | -1.68986 | 0.00188 |
| KEGG_PATHWAY | hsa04080 | Neuroactive ligand-receptor interaction | -1.56677 | 0.001946 |
| KEGG_PATHWAY | hsa05034 | Alcoholism | -1.932 | 0.001972 |
| KEGG_PATHWAY | hsa04360 | Axon guidance | -1.48799 | 0.001976 |
| KEGG_PATHWAY | hsa05203 | Viral carcinogenesis | -1.76108 | 0.001976 |
| KEGG_PATHWAY | hsa05217 | Basal cell carcinoma | -1.94346 | 0.001976 |
| KEGG_PATHWAY | hsa05166 | Human T-cell leukemia virus 1 infection | -1.6042 | 0.00198 |
| KEGG_PATHWAY | hsa04613 | Neutrophil extracellular trap formation | -1.90047 | 0.001992 |
| KEGG_PATHWAY | hsa05202 | Transcriptional misregulation in cancer | -1.51095 | 0.001992 |
| KEGG_PATHWAY | hsa04024 | cAMP signaling pathway | -1.50604 | 0.002 |
| KEGG_PATHWAY | hsa00062 | Fatty acid elongation | -2.0021 | 0.002004 |

**Table 8. GSVA analysis of GSE80178**

| **Category** | **Description** | **p-value** |
| --- | --- | --- |
| KEGG_PATHWAY | KEGG_MATURITY_ONSET_DIABETES_OF_THE_YOUNG | 0.001159 |
| KEGG_PATHWAY | KEGG_RNA_DEGRADATION | 0.001164 |
| KEGG_PATHWAY | KEGG_HOMOLOGOUS_RECOMBINATION | 0.001166 |
| KEGG_PATHWAY | KEGG_LYSINE_DEGRADATION | 0.00117 |
| KEGG_PATHWAY | KEGG_VEGF_SIGNALING_PATHWAY | 0.0012 |
| KEGG_PATHWAY | KEGG_RIBOFLAVIN_METABOLISM | 0.001211 |
| KEGG_PATHWAY | KEGG_DRUG_METABOLISM_CYTOCHROME_P450 | 0.001212 |
| KEGG_PATHWAY | KEGG_GALACTOSE_METABOLISM | 0.001224 |
| KEGG_PATHWAY | KEGG_STEROID_BIOSYNTHESIS | 0.001225 |
| KEGG_PATHWAY | KEGG_VALINE_LEUCINE_AND_ISOLEUCINE_DEGRADATION | 0.001253 |
| KEGG_PATHWAY | KEGG_BASE_EXCISION_REPAIR | 0.001383 |
| KEGG_PATHWAY | KEGG_GLYCOLYSIS_GLUCONEOGENESIS | 0.001391 |
| KEGG_PATHWAY | KEGG_TYPE_II_DIABETES_MELLITUS | 0.00155 |
| KEGG_PATHWAY | KEGG_ONE_CARBON_POOL_BY_FOLATE | 0.002304 |
| KEGG_PATHWAY | KEGG_FRUCTOSE_AND_MANNOSE_METABOLISM | 0.002494 |
| KEGG_PATHWAY | KEGG_NEUROACTIVE_LIGAND_RECEPTOR_INTERACTION | 0.002747 |
| KEGG_PATHWAY | KEGG_ETHER_LIPID_METABOLISM | 0.003077 |
| KEGG_PATHWAY | KEGG_SPLICEOSOME | 0.003135 |
| KEGG_PATHWAY | KEGG_OLFACTORY_TRANSDUCTION | 0.003205 |

**Table 9. GSVA analysis of GSE143735**

| **Category** | **Description** | **p-value** |
| --- | --- | --- |
| KEGG_PATHWAY | KEGG_TASTE_TRANSDUCTION | 0.007742 |
| KEGG_PATHWAY | KEGG_GLYCOSAMINOGLYCAN_BIOSYNTHESIS_CHONDROITIN_SULFATE | 0.008102 |
| KEGG_PATHWAY | KEGG_PRIMARY_IMMUNODEFICIENCY | 0.010052 |
| KEGG_PATHWAY | KEGG_P53_SIGNALING_PATHWAY | 0.012455 |
| KEGG_PATHWAY | KEGG_CELL_ADHESION_MOLECULES_CAMS | 0.016829 |
| KEGG_PATHWAY | KEGG_BASAL_CELL_CARCINOMA | 0.021437 |
| KEGG_PATHWAY | KEGG_SPHINGOLIPID_METABOLISM | 0.026368 |
| KEGG_PATHWAY | KEGG_PENTOSE_AND_GLUCURONATE_INTERCONVERSIONS | 0.033409 |
| KEGG_PATHWAY | KEGG_SYSTEMIC_LUPUS_ERYTHEMATOSUS | 0.033857 |
| KEGG_PATHWAY | KEGG_OTHER_GLYCAN_DEGRADATION | 0.034032 |
| KEGG_PATHWAY | KEGG_ARACHIDONIC_ACID_METABOLISM | 0.034901 |
| KEGG_PATHWAY | KEGG_T_CELL_RECEPTOR_SIGNALING_PATHWAY | 0.036103 |
| KEGG_PATHWAY | KEGG_NOTCH_SIGNALING_PATHWAY | 0.036315 |
| KEGG_PATHWAY | KEGG_OLFACTORY_TRANSDUCTION | 0.044328 |
| KEGG_PATHWAY | KEGG_INTESTINAL_IMMUNE_NETWORK_FOR_IGA_PRODUCTION | 0.047257 |
